# Review of the 2012 Epizootic Hemorrhagic Disease Outbreak in Domestic Ruminants in the United States

**DOI:** 10.1371/journal.pone.0133359

**Published:** 2015-08-05

**Authors:** G. Stevens, B. McCluskey, A. King, E. O’Hearn, G. Mayr

**Affiliations:** 1 U.S. Department of Agriculture, Animal and Plant Health Inspection Service, Veterinary Services, Lincoln, Nebraska, United States of America; 2 U.S. Department of Agriculture, Animal and Plant Health Inspection Service, Veterinary Services, Fort Collins, Colorado, United States of America; 3 U.S. Department of Agriculture, Animal and Plant Health Inspection Service, Veterinary Services, Jefferson City, Missouri, United States of America; 4 U.S. Department of Agriculture, Animal and Plant Health Inspection Service, Veterinary Services, Foreign Animal Disease Diagnostic Laboratory, Plum Island, New York, United States of America; Friedrich-Loeffler-Institut, GERMANY

## Abstract

An unusually large number of cases of Epizootic hemorrhagic disease (EHD) were observed in United States cattle and white-tailed deer in the summer and fall of 2012. USDA APHIS Veterinary Services area offices were asked to report on foreign animal disease investigations and state diagnostic laboratory submissions which resulted in a diagnosis of EHD based on positive PCR results. EHD was reported in the following species: cattle (129 herds), captive white-tailed deer (65 herds), bison (8 herds), yak (6 herds), elk (1 herd), and sheep (1 flock). A majority of the cases in cattle and bison were found in Nebraska, South Dakota, and Iowa. The majority of cases in captive white-tailed deer were found in Ohio, Iowa, Michigan, and Missouri. The most common clinical sign observed in the cattle and bison herds was oral lesions. The major observation in captive white-tailed deer herds was death. Average within-herd morbidity was 7% in cattle and bison herds, and 46% in captive white-tailed deer herds. The average within-herd mortality in captive white-tailed deer herds was 42%.

## Introduction

Epizootic hemorrhagic disease (EHD) is a noncontagious, vector-borne disease transmitted by biting midges of the genus *Culicoides*. Epizootic hemorrhagic disease virus (EHDV) is in the family *Reoviridae* and genus *Orbivirus*. The normal host range of EHDV is wild and domestic ruminants. The distribution of EHDV in the United States is determined by the distribution of *Culicoides sonorenis*, the primary vector of EHDV in North America [1]. *C*. *sonorenis* populations are found in the western, south central, mid-Atlantic, and southeastern United States [2]. EHDV has also been identified in Australia, Africa, and Asia [3–5]. Mertens described 10 serotypes of EHDV: serotypes 1–8, EDHV-318, and Ibaraki virus [6]. However, it has been proposed these 10 serotypes be condensed down to 7 serotypes [7]. The serotypes of EHDV which have been identified in the United States are EHDV-1, EHDV-2, and EHDV-6 [8–11].

EHD was first described in 1955 in a New Jersey outbreak characterized by high mortality in white-tailed deer [8]. Outbreaks in white-tailed deer are seasonal, occurring from mid-summer to late autumn and appear to occur every 2 to 3 years in endemic areas, and every 8 to 10 years in epidemic areas [12–13]. Three disease syndromes have been described in white-tailed deer: peracute, acute, and chronic. Clinical signs can range from rapid death in the peracute syndrome to sloughing of hooves in the chronic syndrome. Other clinical signs may include fever, anorexia, respiratory distress, edema of the head and neck areas, excessive salivation, oral erosions, and lameness [14].

EHDV infection in cattle does not usually result in clinical disease. When seen, it is a less severe disease than what is observed in deer. Clinical signs observed in cattle may include fever, anorexia, loss in milk production, swollen conjunctiva, nasal and ocular discharge, excessive salivation, stomatitis, oral and nasal erosions, lameness, and dyspnea [15–16]. Laboratory-confirmed cases of EHD have been reported in Oregon in 1969, Tennessee in 1972, and Colorado in 1974 [15]. A study of investigations of cattle with vesicular lesions completed in late summer and early fall in 1996 in 9 Midwestern states reported 32 premises with animals serologically positive for EHDV [16]. A serological study of cattle in Illinois and Indiana detected antibodies to EHDV in 11.8% of 1,110 cattle tested [17].

The clinical signs of EHD in cattle can be similar to those of bluetongue, bovine viral diarrhea, foot-and-mouth disease (FMD), infectious bovine rhinotracheitis, vesicular stomatitis (VS), malignant catarrhal fever (MCF), and bovine ephemeral fever. Because the clinical signs of EHD in cattle are similar to those seen in FMD, VS, and MCF, these cases are often referred to state and federal animal health authorities for investigation as possible foreign animal diseases.

An unusually large number of cases of EHD were observed in United States cattle in summer and fall of 2012. The largest numbers of cases were reported in Iowa, Nebraska, and South Dakota. This coincided with a large number of reported cases in captive and free-ranging white-tailed deer in some regions of the United States. The purpose of this paper is to report the findings of foreign animal disease investigations, state veterinary diagnostic laboratory submissions, and other investigations which resulted in a diagnosis of EHD in domestic ruminants.

## Material and Methods

Biological samples for foreign animal disease investigations were collected by veterinarians employed by USDA APHIS Veterinary Services or State Departments of Agriculture who have received specialized training in the diagnosis of foreign animal diseases. Samples submitted to State Veterinary Laboratories were collected for diagnostic purposes by licensed veterinarians or veterinarians employed by State Departments of Agriculture. The types of samples collected included blood samples (serum or whole blood) collected from the jugular or tail (coccygeal) vein, swab or tissue from oral erosions, vesicular fluid from oral lesions, or tissue samples collected from post-mortem examinations of animals. No sacrifice of animals was conducted.

No protected species were sampled during the course of this study. All animal care and husbandry was provided by livestock owners. Permission to examine and collect samples from livestock was voluntarily granted by their owners. The USDA does not have an Institutional Animal Care and Use Committee (IACUC) with oversight over diagnostic samples collected from privately owned livestock during a foreign animal disease investigation.

Private practitioners or livestock producers who observed vesicular clinical signs in livestock reported these conditions to state or federal animal health officials. A foreign animal disease diagnostician (FADD) was dispatched to examine the livestock and collect diagnostic samples. FADDs are state or federal regulatory veterinarians specifically trained in the diagnosis and sampling methods for diseases foreign to the United States. The findings of the investigation were recorded in the Emergency Management Response System (EMRS), a USDA–APHIS Veterinary Services (APHIS–VS) database used to manage disease investigation and response activities. Diagnostic samples were submitted to the National Veterinary Services Laboratories (NVSL) Foreign Animal Disease Diagnostic Laboratory (FADDL) on Plum Island, New York. After foreign animal diseases were ruled out, samples were tested by the EHD reverse transcription polymerase chain reaction assay (RT-PCR).

EHD real-time RT-PCR was performed using a previously published assay which detects the 8 prototype serotypes but was modified to include only 1 primer and probe set as follows [18]. RNA was isolated from whole EDTA blood using QIAampViral RNA extraction kit (QIAGEN, Valencia, CA). EHD RNA (2.5 μL) was mixed with 1 μL of 10 μM forward and reverse primers and 8 μL of water and denatured at 95°C for 5 min followed by a 1-min ice quench. The denatured template (11 μL) was transferred into a master mix with 14 μL of AgPAth-ID one-step RT-PCR reagents (Life Technologies, Carlsbad, CA). The master mix contained 12.5 μL 2X RT-PCR buffer, 1 μL 25X enzyme mix, and 0.5 μL of 10 μM probe labeled with 5’FAM and 3’BHQ-1 (Biosearch Technologies, Inc, Petaluma, CA). Real-time amplification was performed on a SmartCycler II thermal cycler (Cepheid Inc., Sunnyvale, CA) using the following cycling conditions: 1 cycle of 48°C for 1400 sec and 95°C for 900 sec, followed by 40 cycles of 95°C for 15 sec, 48°C for 40 sec, and 70°C for 20 sec. The primers and probe were designed from EHD S10 gene, encoding the nonstructural protein NS3, and targeting EHD serotypes 1, 2, 5, 6, and 7:

EHD Probe 192: 5’FAM-TCAAATCAAACGGGCGCAACTATGG-3’BHQ-1

EHD Forward Primer 165: 5’-GCGTTGGATATATTGGACAAAGC-3’

EHD Reverse Primer 253: 5’-GCATACGAAGCATAAGCAACCTT-3’

All APHIS–VS area offices were asked to submit a set of structured standardized data on FAD investigations resulting in a diagnosis of EHD conducted between June 1 and December 31, 2012. Table 1 summarizes the data elements collected.

**Table 1:** Data requested from USDA–APHIS Veterinary Services Area Offices on foreign animal disease investigations and state diagnostic lab submissions between June 1 and December 31, 2012.

Accession or referral number	^1^ Clinical symptoms	Diagnostic testing results
County	Fever	EHD AGID
^1^Address, City, State	Off Feed Difficulty Eating	EHD PCR
^1^Latitude and Longitude	Excessive Salivation	Other EHD test
Investigation or collection date	Muzzle Lesions	Bluetongue ELISA
Species affected	Oral Lesions	Bluetongue PCR
^1^Date of onset	Teat Lesions	Other BT test
^1^Nr sick, Nr dead, Nr unaffected	Lameness/Stiffness	EHD serotype

^1^ Not required from state laboratories; data may be confidential or was not available.

### Collection of data from state laboratories

Not all EHD cases were managed as FAD investigations. APHIS–VS offices were also asked to request data from state diagnostic laboratories on submissions with positive EHD results in domestic ruminants with clinical signs suggestive of EHD. Table 1 summarizes the data elements that were requested on submissions to state laboratories.

### Collection of data from STRAND

EHD results for testing conducted at NVSL and FADDL were extracted from the Searchable Test Results Application for NVSL Diagnostics (STRAND) database for the period June 1 to December 31, 2012. These data were used to validate test results for the FAD investigations and to validate state diagnostic lab test results in cases where positive samples were forwarded to NVSL for serotyping. Additional cases were identified though they were not submitted as a result of the data request. Accessions were excluded from the data analysis if one or more of the following conditions were true: 1) results did not meet the case definition (PCR positive), 2) results were already reported through a state lab or foreign animal disease investigation, 3) the species was not a domestic ruminant, 4) the samples were collected from animals outside the United States, or 5) the testing was for movement of animals or certification of germplasm. Any possible new cases identified from the STRAND database were confirmed by contacting the APHIS–VS Area Office for the state where the animals were located.

### Collection of data from clinical cases in Missouri captive white-tailed deer

A particularly large number of EHD cases occurred in both Missouri wild and captive white-tail deer populations during the last half of 2012. Reporting of EHD occurrence in captive cervid herds to the Missouri state animal health authorities is not mandatory. In an attempt to capture this information, personal contacts were made with 19 herd veterinarians (or their clinics) working with most of the captive cervid herds in Missouri’s Tuberculosis Accredited-Free herd program. There were approximately 105 herds in the program, and the veterinarians contacted represented 90 of those herds. The veterinarians were asked about the occurrence of clinical signs, similar to the information gathered in cattle herds, as well as herd morbidity and mortality. Information was eventually gathered for 49 of Missouri’s captive cervid herds. All of the data captured were for white-tailed deer. Because of the prevalence of EHD, the cost of testing, and the peracute nature of the disease, testing had been conducted in only 12 of these 49 herds. The rest of the herds were diagnosed on clinical signs and morbidity/mortality levels without testing any samples.

### Data analysis

A case was defined as a premises which had animals with clinical signs suggestive of EHD and which had EHDV detected by PCR analysis. The case date (week) for use in constructing the epidemic curve was determined using the sample collection date or lab submission date. When this was not available, the reported date of onset was used. The case date for the Missouri captive white-tailed deer cases diagnosed based on clinical signs was the investigation date. A within-herd morbidity rate was calculated by the following formula: $Within-herd\;morbidity=\left(\frac{Nr\;Sick+Nr\;Dead}{Nr\;Sick+Nr\;Dead+Nr\;Unaffected}\right)\times 100$. A within-herd mortality rate was calculated by the following formula: $Within-herd\;mortality=\left(\frac{Nr\;Dead}{Nr\;Sick+Nr\;Dead+Nr\;Unaffected}\right)\times 100$. The abbreviation “Nr” refers to the number of animals entered into this formula. Each investigation was evaluated to determine if it met the case definition. Investigations which did not meet the case definition were excluded from the analysis.

## Results

**Fig 1:**
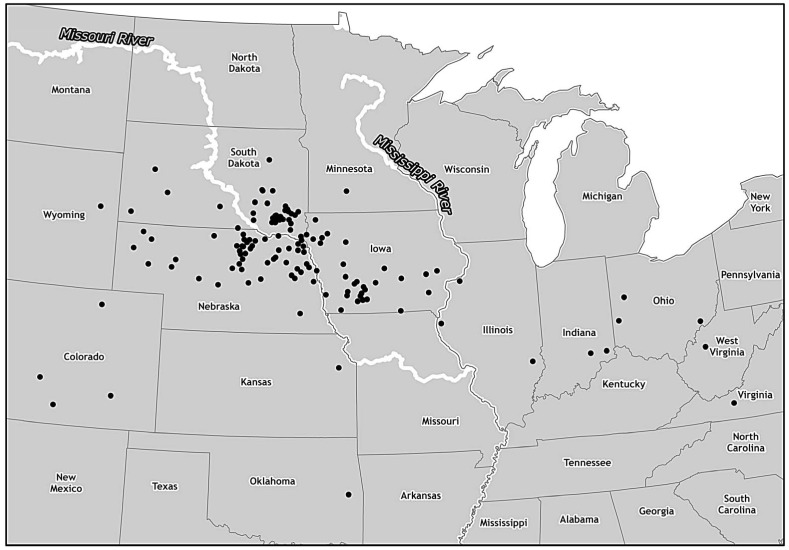
Density of bovine EHD cases during 2012 outbreak. To protect the confidentiality of individual producers, each symbol represents a bovine case of EHD randomly placed in the county in which it occurred.

EHD cases resulting from FAD investigations of vesicular conditions in cattle and bison were reported from 12 states. Eighty cases were reported in cattle and 3 in bison. Nebraska (46 cases) and Iowa (22 cases) reported the most cases of EHD resulting from FAD investigations. EHD-positive cases resulting from submissions to state diagnostic laboratories were reported from 7 states. Forty-nine cases were reported in cattle and 5 cases were reported in bison. South Dakota (36 cases) and Iowa (9 cases) reported the most cases resulting from submissions to state laboratories. Overall, 15 states reported EHD cases from FAD investigations or state laboratory submissions. One hundred twenty-nine cases were reported in cattle and 8 cases were reported in bison. Nebraska (49 cases), South Dakota (37 cases), and Iowa (31 cases) reported the most cases in cattle and bison. EHD cases in cattle and bison by state are summarized in Table 2. The locations of cases in cattle and bison are illustrated in Fig 1.

**Table 2:** EHD cases in cattle and bison from June 1 to December 31, 2012.

	Foreign animal disease investigations			Submissions to state laboratories			Total cases
State	Bison	Bovine	Total	Bison	Bovine	Total	
Colorado		3	3		1	1	4
Iowa		22	22	1	8	9	31
Illinois					3	3	3
Indiana		2	2				2
Kansas		1	1				1
Maryland		1	1				1
Minnesota		1	1				1
Nebraska	2	44	46		3	3	49
Ohio	1	2	3				3
Oklahoma		1	1				1
Pennsylvania		1	1				1
South Dakota		1	1	4	32	36	37
Virginia					1	1	1
West Virginia					1	1	1
Wyoming		1	1				1
Total	3	80	83	5	49	54	137

**Fig 2:**
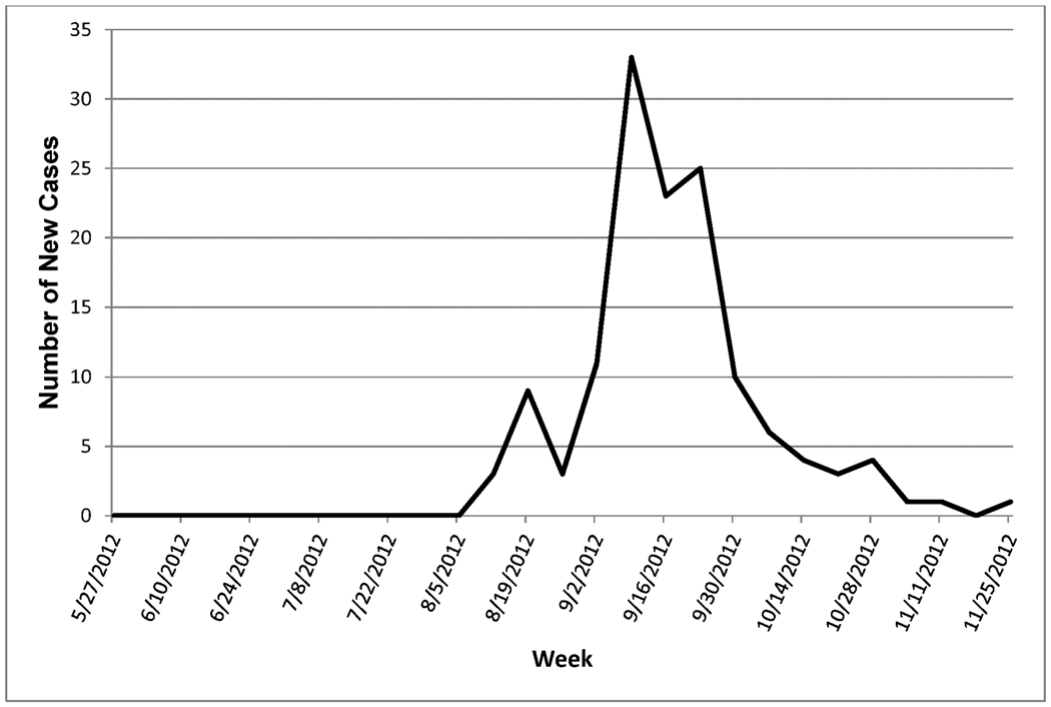
Number of new EHD cases in cattle and bison by week.

The first case of EHD in cattle or bison was reported during the week of August 12, 2012, in Nebraska. The last case was reported during the week of November 25, 2012, in South Dakota. The majority of the cases (74%) were reported between the weeks of September 2 and September 30, 2012. Fig 2 summarizes the number of EHD cases by week in cattle and bison.

Clinical signs were reported for 85 of the EHD cases in cattle and bison. The most common sign reported was oral lesions. Overall, 86% of the case-positive premises reported at least 1 animal with oral lesions. Other clinical signs reported in at least 1 animal on case-positive premises were excessive salivation (64%), off feed or difficulty eating (57%), lameness/stiffness (49%), muzzle lesions/crusty muzzle (47%), fever (32%), and teat lesions (7%).

Complete information on the number of animals affected and number of susceptible animals was recorded for 82 of the cases (3 bison and 79 cattle). The average within-herd morbidity was 7.12%. The median within-herd morbidity was 3.23%. The reported within-herd morbidity ranged from 0.05% to 100.00%. Overall, 70 of 82 cases reported a within-herd morbidity rate of 10% or less. Of 96 cases which recorded the number of affected animals, 66 cases (69%) reported only 1 animal with clinical signs. Four cases, 2 bison and 2 cattle, had mortality due to EHD.

**Fig 3:**
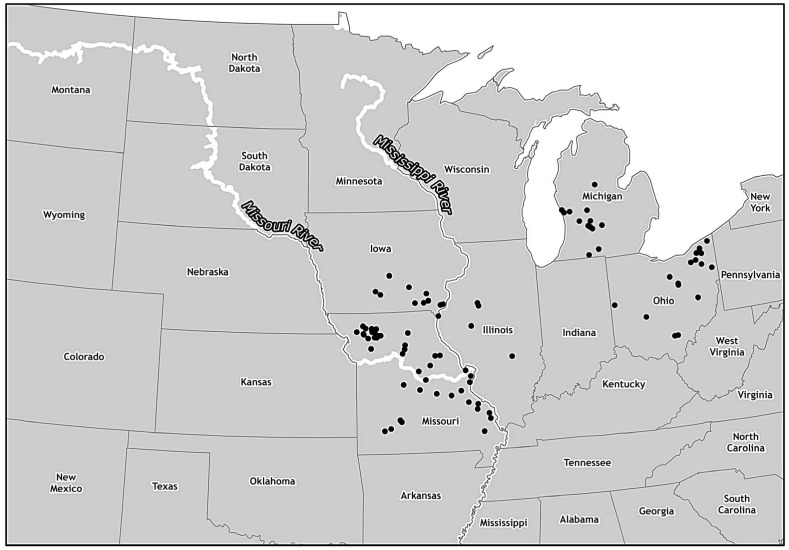
Density of captive white-tailed deer EHD cases during 2012 outbreak. To protect the confidentiality of individual producers, each symbol represents a captive white-tailed deer case of EHD randomly placed in the county in which it occurred.

Eleven states reported a total of 65 EHD cases in captive white-tailed deer identified through submissions to state diagnostic laboratories. Ohio (16 cases), Michigan (13 cases), and Iowa (13 cases) reported the most cases resulting from submissions to state laboratories. Missouri reported 9 cases resulting from submissions to state diagnostic laboratories. Missouri also reported 34 cases in white-tailed deer based on clinical signs as described in interviews with private practitioners. EHD cases in captive white-tailed deer by state are summarized in Table 3. The locations of cases in captive white-tailed deer are illustrated in Fig 3.

**Table 3:** EHD cases in white-tailed deer from June 1 to December 31, 2012.

State	Missouri clinical cases (No lab)	Submissions to state laboratories	Total
Alabama		1	1
Florida		2	2
Iowa		12	12
Illinois		4	4
Louisiana		1	1
Michigan		13	13
Missouri	34	9	43
North Carolina		2	2
New Jersey		1	1
Ohio		16	16
Texas		4	4
Total	34	65	99

**Fig 4:**
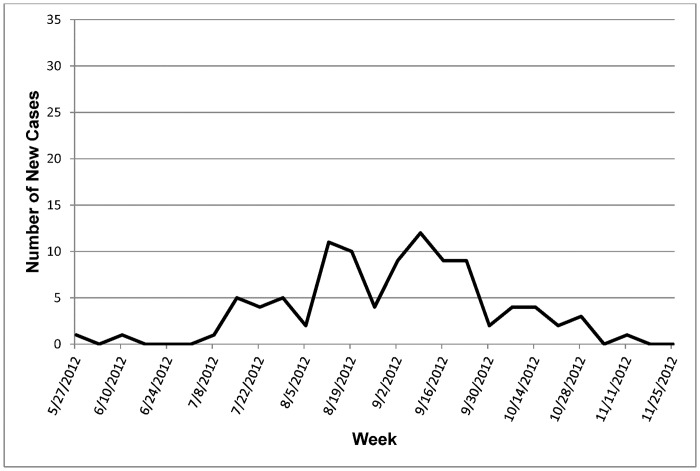
Number of new EHD cases in captive white-tailed deer by week.

The first case of EHD in captive white-tailed deer was reported on June 1, 2012, in Louisiana, which was the beginning date for this study’s data request. The last case was reported during the week of November 13, 2012, based on a clinical diagnosis in Missouri. The majority of the cases (65%) were reported between the weeks of August 12 and September 23, 2012. Fig 4 summarizes the number of EHD cases by week.

Clinical signs were reported for 52 of the EHD cases in captive white-tailed deer. Mortality due to EHD was reported in at least 1 animal on 46 (88%) of the case-positive premises. The most common clinical signs reported in at least 1 animal on the case-positive premises were: being off feed (90%) and fever (81%). Other clinical signs reported in at least 1 animal were: excessive salivation (58%), lameness (38%), oral lesions (17%), muzzle lesions/crusty muzzle (15%), and teat lesions (2%).

Complete information on the number of white-tailed deer affected and number of susceptible animals was reported for the 43 clinical and laboratory cases in Missouri. The average within-herd morbidity was 46%. The median within-herd morbidity was 47%. The reported within-herd morbidity ranged from 3% to 94%. The average within-herd mortality was 42%. The median within-herd mortality was 38%. The reported within-herd mortality ranged from 3% to 84%.

Events meeting the EHD case definition were also reported in 6 yak herds in Colorado [19], 1 elk herd in Iowa, 1 sheep flock in Iowa, and 1 deer herd in Florida.

## Discussion

An appreciation of the magnitude of the EHD outbreak in 2012 is realized by examining the results of FAD investigations conducted in previous years. During late summer and fall in 2012, there were 83 FAD investigations in cattle and bison resulting in a diagnosis of EHD based on a positive RT-PCR test. During 2010–2011, 3 FAD investigations in the United States resulted in a diagnosis of EHD based on positive PCR results (NVSL STRAND). It is likely that EHD cases were under-reported in areas with high EHDV activity as veterinarians and producers started diagnosing EHD based on clinical signs. This is evident from the data submitted from Missouri on EHD cases in their captive cervid industry. Missouri reported over 3 times the cases based only on clinical symptoms than based on lab results. Other states with captive white-tailed deer operations also remarked that the laboratory cases did not represent the number of cases observed in their respective captive cervid industries.

Most of the data on affected animals and clinical signs were compiled from FAD investigations. It should be noted that in most cases not all animals within a herd were examined or tested for EHD. Therefore, the actual morbidity rates within a herd may be higher than the morbidity rate reported here. In addition, there is no standardized format for recording specific clinical signs in EMRS. Therefore, some of the less dramatic clinical signs, such as fever or being off feed, may not always have been recorded by the FADD. Reporting of cases from state diagnostic laboratories was voluntary and reported through the respective area office. Reports were received from 48 of 50 states. Difficulty with interpreting state diagnostic laboratory data may have involved differentiating cases involving captive white-tailed deer versus free-ranging white-tailed deer and differentiating submissions from clinical animals versus animals tested for other purposes. However, these difficulties presented in states with minimal expected EHD activity and did not have a bearing on the findings of this study.

We also requested general information on EHD serotypes which were identified during these investigations. While serotyping was not the focus of this study, we feel these results are still worthwhile to include in this report. However, because EHD serotyping was not completed on all laboratory submissions, the serotypes reported below may not be completely representative of what occurred during this outbreak.

The EHD serotype was determined for 59 of the 133 cases of EHD in bison and cattle. Serotype 2 was identified in 54 of these cases, which were located in Nebraska, Iowa, South Dakota, Minnesota, and Ohio. Serotype 6 was identified in 3 cases. These cases were located in Illinois and Iowa. Serotype 1 was identified in 1 South Dakota case. One Illinois case in cattle identified serotype 2 and serotype 6 on the same premises.

The EHD serotype was determined for 33 of the cases of EHD in captive white-tailed deer. Serotype 2 was identified in 21 of these cases, which were located in Iowa, Missouri, New Jersey, North Carolina, Ohio, and Texas. Serotype 6 was identified in 8 cases in Florida, Illinois, and Iowa. Serotype 1 was identified in 3 cases, located in Florida, Texas, and Louisiana. One Missouri case in captive white-tailed deer identified serotype 2 and serotype 6 on the same premises.

It is likely that the high temperatures associated with drought conditions present in many states in 2012 contributed to this EHD outbreak. The National Climatic Data Center reported that 2012 was the warmest year in the 1895–2012 period of record for the contiguous United States [20]. Nineteen states, including Nebraska, South Dakota, and Missouri, had record warm annual temperatures in 2012. High temperatures facilitate the transmission of EHD virus by C. sonorensis midges by decreasing the extrinsic incubation period of the virus [21].

A previous study conducted in Nebraska, South Dakota, and North Dakota indicated that the risk of cattle being seropositive to bluetongue virus (BTV), another orbivirus transmitted by *C*. *sonorensis* midges, decreased at more northerly latitudes. This study indicated that BTV was endemic in southern Nebraska [22]. This study also indicated that the eastern limit of *C*. *sonorensis* in South Dakota is clearly defined by the location of the Missouri River, and that the eastern limit in Nebraska may also be the Missouri River [23]. In South Dakota and Iowa, a majority of the EHD cases were seen in areas outside the normal range of *C*. *sonorensis*. Thirty-two of the 37 cases in South Dakota were in counties east of the Missouri River. A majority of the cases in Iowa were seen in the western area of the state. Iowa is not considered to be in the normal distribution of *C*. *sonorensis*, but western Iowa is adjacent to areas of Nebraska where *C*. *sonorensis* is normally found. In Nebraska, all but 1 of the cases was located in the northern half of the state. The regionalization of confirmed clinical cases in these 3 states indicates expansion from the normal distribution of EHDV. The resulting exposure of cattle and white-tailed deer with limited immunity to EHDV resulted in the increase in number of clinical cases of EHD in cattle and captive white-tailed deer. Whether this expansion of EHDV exposure was due to an actual expansion of the range of *C*. *sonorensis* or an amplification of EHDV within existing *C*. *sonorensis* populations is beyond the scope of this observational study.

The clinical signs of EHD can be similar to those seen with foot-and-mouth disease, vesicular stomatitis, and malignant catarrhal fever. Oral lesions and excessive salivation were seen in a majority of the affected herds. Muzzle lesions and lameness were seen in nearly 50% of the herds. The occurrence of EHD during the vector season and low morbidity rates in cattle are especially similar to vesicular stomatitis. If one of these foreign animal diseases is introduced into an area experiencing an outbreak of EHD, detection could be delayed if the clinical signs are attributed to EHD and if the condition is not investigated as a possible foreign animal disease.
